# Cancer of the Biliary Ducts

**Published:** 1908-09-26

**Authors:** 

**Affiliations:** Paris, specially reported for The Hospital.


### Hospital Clinics.

### CANCER OF THE BILIARY DUCTS.

(From a Clinical Lecture by PROFESSOR DEBOVE, Paris, specially reported for The Hospital.)

Cancer of the biliary ducts presents two essential characteristics: it develops primarily in these ducts and it has very little tendency to become generalised. That such a cancer exists is not to be wondered at, for cancer is an epithelial affection and the biliary ducts are lined with epithelium and contain glands. If this variety of cancer has little tendency to generalise, the reason is that at a very early date the bile ducts become completely blocked, and the existence of the patient compromised; in other words, the affection is too rapid for generalisation to take place.

Cancer may occupy different points in the biliary ducts. It may, first of all, occupy "Vater's ampulla," and there has been a great deal of controversy as to whether in this case it is really a biliary cancer or a cancer of the pancreas, or, again, a cancer of the intestinal mucous membrane situated at the orifice of the biliary and pancreatic ducts. However, this question presents very little interest to the clinician, for the symptoms are always the same, the prognosis is the same, and the therapeutical indications are the same.

If the cancer is seated in the common duct the symptoms are the same as in the foregoing case. There is marked and chronic jaundice, the gall bladder is distended, the liver is enlarged, and very often the spleen is also enlarged. The jaundice is easily explained by the early obliteration of the duct. The distension of the gall bladder is the rule and helps the diagnosis considerably, for this distension does not exist as a rule when the obstruction of the duct is due to a calculus. In the latter case there is nearly always a chronic inflammation of the gall bladder, which gives rise to thickening and retraction of its walls and thus increases its resistance to distension.

The enlargement of the liver is due to distension of the biliary ducts, to congestion, and also perhaps to cirrhosis of the organ. Authorities, however, do not quite agree on the nature of the lesions which accompany hypertrophy of the hepatic gland. In cancer of the gland itself and of the cystic duct the disease nearly always remains latent until the common duct is attacked, and then the symptoms enumerated above are present with the addition of a tumour in the region of the gall bladder. If the cancer is seated in the hepatic duct there is jaundice, but the bladder is not distended. In this variety the liver and the spleen present very little or no hypertrophy.

The foregoing preliminary remarks are necessary in order to understand the evolution of the disease from which the patient whose case is related below was suffering. It was a case of cancer of the hepatic duct near its bifurcation.

A. B.—Æt. 75, was admitted into the hospital on the 23rd of October, and died on the 21st of November. His father and mother died very old. There are four brothers and sisters living. There is no cancer or liver affection in the family. He always enjoyed the best of health, had not had syphilis, and was always most temperate as regards alcohol, his daily allowance being half a litre of wine. He presented no symptom of alcoholism.

About a month before coming to the hospital he noticed that he was yellow and had generalised pruritus. In cancer of the biliary passages the onset is always latent, and jaundice is the first symptom noticed. Pruritus is very frequent in these cases, and its intensity is due to the rapidity and the degree of the jaundice. The patient did his best to continue working, but he soon noticed that his strength diminished, and that he lost weight. He always felt fatigued and had to lie down the greater part of the day. On his entrance into the hospital the first thing that struck one was his marked jaundice. The skin and mucous membranes were a deep yellow. Personally, I have never seen a case in which jaundice was so intense. The urine contained such a quantity of biliary pigments that it was quite black. Gmelin's reaction could only be obtained by greatly diluting the urine, the pigments being so abundant that the reaction was quite distinct when using a mixture of one part of urine and twenty parts of water. The saliva and expectoration contained pigments. The blood serum was of a saffron yellow and very rich in pigments.

The fæces were quite decolourised, showing that the jaundice was due to retention. The examination of the liver was rendered quite easy, owing to the emaciation of the patient; the organ was not enlarged and the gall bladder was not distended. It is important to note that there was neither ascites nor collateral circulation, and that the spleen was not enlarged. The heart was sound, but its action slow. The lungs presented signs of bronchitis and congestion at the bases.

The urine passed in 24 hours amounted to 1 litre; there was no albumin. Tested for alimentary glycosuria, the result was negative. The amount of urea excreted in 24 hours was about 15 grammes.

The nervous symptoms were very marked, asthenia being so intense that the patient remained on his back all day long and had to be supported when he wished to sit up. There was a continual state of drowsiness, although he roused up every now and then to scratch himself. The temperature in the axilla was 37° Centigrade. The appetite was poor, no vomiting nor diarrhœa, although he had lost 12 kilogrammes in one month.

After our examination we made the diagnosis of cancer compressing the biliary ducts without defining the cause more fully, and I wish to show that we might have done so, and diagnosed by exclusion a cancer of the biliary ducts.

It was quite clear that it was a case of jaundice by retention; the discoloured fæces and the intensity of the jaundice were quite sufficient to show this. The cause of the obstruction was not a calculus, for the patient had never had any hepatic colic nor any pain which might have simulated it; besides, the degree of the jaundice, the rapidity of its onset, the early collapse of the general state of health were not in favour of such a diagnosis. I do not discuss the hypothesis of biliary cirrhosis, for there was no hypertrophy of either the liver or spleen, and the fæces were permanently discoloured. The general state of the patient and rapid loss of weight were in favour of cancer. The idea of cancer of the stomach or of the bowels compressing the biliary ducts could be eliminated, for there had never been any symptoms of gastric or intestinal trouble.

Was it a case of cancer of the head of the pancreas or of "Vater's ampulla"? For this diagnosis two important symptoms were missing; there was no enlargement of the liver and no distension of the gall bladder. We could therefore have diagnosed a cancer of the hepatic duct, especially by taking into account the intensity of the jaundice, which I believe to be more marked in this variety of cancer of the biliary ducts than in any other.

The patient remained four weeks in our ward before dying. During the three first weeks he presented no new symptoms, but during the last week he presented a series of symptoms which are habitually associated with acute yellow atrophy of the liver and which may be classified as follows:—

1. *Multiple hæmorrhage;* bleeding from the gums, abundant and repeated epistaxis, large purpuric spots on the body and limbs.

2. *Diminution in the quantity of the urine,* which fell to 250, 200, and even 100 grammes in 24 hours. This diminution was certainly due to general causes, to a fall of the blood pressure, to lesions of the kidneys, the quantity of urea eliminated in 24 hours being only a few grammes, and the alimentary glycosuria test, which had been negative at first, becoming positive during the last few days.

3. *Accentuation of the nervous symptoms;* the patient presenting attacks of delirium, followed by periods of coma, during which he responded to no excitation, and simply muttered a few incoherent words.

4. *Pigmentary acholia;* during the last few days of the patient's life the urine no longer gave Gmelin's reaction, and the blood serum recovered its normal colour. Perhaps nothing can show so clearly the deep disorganisation of the hepatic functions as this symptom. It showed that biliary pigments were no longer manufactured; if the skin still retained its yellow colour it was simply due to the intensity of its impregnation.

5. *Hypothermia;* the temperature falling gradually to 36.2 and finally to 35.5 C. This fall of the temperature was a bad sign, and all the more so since the pulse was increasing in rapidity, attaining 150 on the day of the patient's death.

The post-mortem showed cancerous lesions, strictly limited to the hilum of the liver. The only lesion found in the other organs was slight hypostatic congestion at the bases of the lungs; the pancreas, Vater's ampulla and the common duct were normal. The spleen also was quite normal, both as regards volume and consistency. The gall bladder was very much retracted and thickened, and contained only a little mucus; microscopically it presented no cancerous lesions whatsoever. The cystic duct contained no calculi, and was not obliterated. The hepatic duct and its two branches, however, were completely blocked, owing to the fact that the duct at its point of entrance in the liver was situated in the centre of a mass of cancerous tissue, and having undergone itself neoplastic degeneration. The cancerous mass formed a tumour of the size of a small orange; it was firm and whitish, forming a marked contrast to the rest of the liver, stained green as it was with bile. There were no other cancerous deposits either in the liver or in any other organ. Microscopically the tumour was a cylindrical celled epithelioma, the shape of the cells seeming to indicate that they sprang from the epithelial cells of the biliary ducts.

It is interesting, from the point of view of prognosis, to differentiate cases such as this one, which end fatally with great rapidity, from other cases of chronic jaundice, in which the prognosis is much less serious. If obstruction by a calculus or by a cancer of the head of the pancreas had been diagnosed, surgical treatment might have been attempted, such as cholecystenterostomy or cholecystotomy. In the case related surgical treatment would have been absolutely useless and dangerous, for as no bile could get into the gall bladder, it would have been absurd to make the latter communicate with the bowel. On the other hand, in cases of obstruction by calculi surgical treatment applied in proper time has often saved a patient's life.

